# Effective relief of neuropathic pain by adeno-associated virus-mediated expression of a small hairpin RNA against GTP cyclohydrolase 1

**DOI:** 10.1186/1744-8069-5-67

**Published:** 2009-11-18

**Authors:** Sung Jin Kim, Won Il Lee, Yoon Sun Lee, Dong Hou Kim, Jin Woo Chang, Seong Who Kim, Heuiran Lee

**Affiliations:** 1 Departments of Microbiology, University of Ulsan College of Medicine, Seoul, Korea; 2 Biochemistry & Molecular Biology, University of Ulsan College of Medicine, Seoul, Korea; 3 Anatomy & Cell Biology, University of Ulsan College of Medicine, Seoul, Korea; 4 Research Institute for Biomacromolecules, University of Ulsan, Seoul, Korea; 5 Department of Neurosurgery, Yonsei University College of Medicine, Seoul, Korea

## Abstract

**Background:**

Recent studies show that transcriptional activation of GTP cyclohydrolase I (GCH1) in dorsal root ganglia (DRG) is significantly involved in the development and persistency of pain symptoms. We thus hypothesize that neuropathic pain may be attenuated by down-regulation of GCH1 expression, and propose a gene silencing system for this purpose.

**Results:**

To interrupt GCH1 synthesis, we designed a bidirectional recombinant adeno-associated virus encoding both a small hairpin RNA against GCH1 and a GFP reporter gene (rAAV-shGCH1). After rAAV-shGCH1 was introduced into the sciatic nerve prior to or following pain-inducing surgery, therapeutic efficacy and the underlying mechanisms were subsequently validated in animal models. The GFP expression data indicates that rAAV effectively delivered transgenes to DRG. Subsequently reduced GCH1 expression was evident from immunohistochemistry and western-blotting analysis. Along with the down-regulation of GCH1, the von Frey test correspondingly indicated a sharp decline in pain symptoms upon both pre- and post-treatment with rAAV-shGCH1. Interestingly, GCH1 down-regulation additionally led to decreased microglial activation in the dorsal horn, implying an association between pain attenuation and reduced inflammation.

**Conclusion:**

Therefore, the data suggests that GCH1 levels can be reduced by introducing rAAV-shGCH1, leading to pain relief. Based on the results, we propose that GCH1 modulation may be developed as a clinically applicable gene therapy strategy to treat neuropathic pain.

## Background

Neuropathic pain is a chronic disease caused by aberrant pathologic features originating from tissue damage or inflammation within related nerve systems [1]. Typical symptoms include spontaneous pain, exaggerated response to noxious stimuli (hyperalgesia) and pain in response to normally innocuous stimuli (allodynia). A number of therapeutic drugs, such as non-steroidal drugs (NSAIDs) and opioids, have been introduced with the purpose of relieving symptoms [2]. Unfortunately, a significant proportion of drug-treated patients still suffer from pain symptoms, mainly due to a lack of response to drugs [3,4]. Moreover, built-up tolerance to currently available drugs has been frequently reported among patients. Thus, rapid development of alternative therapeutic strategies for these 'difficult-to-treat' pain symptoms is essential.

A number of studies consistently suggest that gene-based therapy holds promise as an alternative approach for treating pain [5-7]. GTP cyclohydrolase I (GCH1) is a rate-limiting enzyme in tetrahydrobiopterin (BH4) synthesis, an essential cofactor for nitric oxide synthase [8]. GCH1 transcription is activated in dorsal root ganglia (DRG) immediately after nerve injury [9]. Moreover, the enzyme plays a crucial role in pain sensitivity and maintenance [10]. One of the most significant findings of this study is the direct linkage of GCH1 expression alterations as underlying phenomena in animal models to genotypic characteristics within the human population. Indeed, healthy individuals with the GCH1 haplotype experienced reduced GCH1 activation and lowered pain sensitivity. These results suggest that GCH1 down-regulation is a promising strategy to treat neuropathic pain with minimal side-effects, which subsequently suggests the importance of developing an effective gene silencing system.

RNA interference (RNAi) is a proven selective gene silencing technique for promoting sequence-specific degradation of complementary RNA [11,12]. A double-stranded small interfering RNA (siRNA) about 21 nucleotides in length is identified as the effector molecule. Several studies have confirmed the therapeutic potential of synthetic siRNA-based strategies in neurodegenerative diseases [2,13,14]. To sustain pain-attenuating effects via the down-regulation of target genes, substantial levels of siRNA are essential. However, gene knockdown effects only last for a few days, largely due to the rapid decay of siRNA within cells [15]. Paddison and colleagues suggested the possibility of intracellular siRNA synthesis for longer lasting gene regulating effects by simply introducing a short-hairpin RNA (shRNA) construct into cells encoding corresponding siRNA information under control of the endogenous RNA polymerase promoter region [16]. In addition, recombinant adeno-associated virus (rAAV), particularly serotype 2 (rAAV2), is an excellent gene delivery vehicle for long-term transgene expression in post-mitotic neural cells [17,18]. Using a rat animal model, we previously demonstrated that rAAV2 induces therapeutic gene expression in DRG and subthalamic nucleus in the brain over several months [19,20]. In this regard, rAAV is a promising vector for the development of gene-based therapy for neuropathic pain.

In the present study, we constructed a rAAV2 harboring the shRNA sequence corresponding to GCH1 siRNA to validate its therapeutic potential in the treatment of pain symptoms. Using an animal pain model, we provide evidence that downregulation of GCH1 by shGCH1 via rAAV delivery is readily achieved, and leads to relief of neuropathic pain. Accordingly, we propose that suppression of GCH1 using rAAV is a promising gene-based strategy to treat chronic pain.

## Results

### Effects of gene silencing of rat GTP cyclohydrolase (rGCH1) with siRNA and shRNA

**Figure 1:**
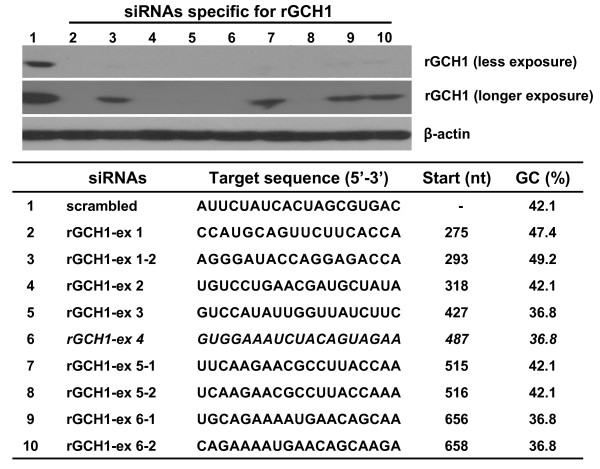
**siRNAs specific for GCH1 and their gene silencing effect**. In the presence of rGCH1 expression vector (pcDNA3-rGCH1), 293T cells were transfected either scrambled control or rGCH1-specific various siRNAs. Two days after transfection, cells were harvested and western-blotting was carried out. The data revealed a significant decrease in the rGCH1 level is observed when siRNAs for rGCH1 are present.

**Figure 2:**
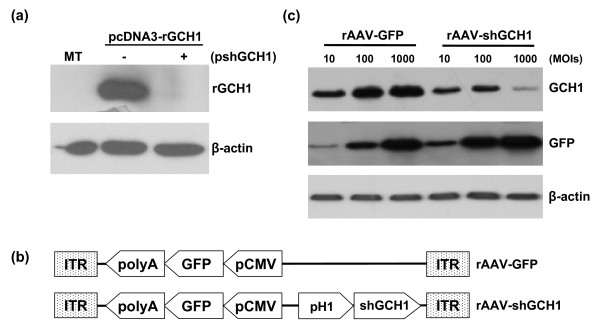
**rAAV constructs and its gene silencing effect**. (a) 293T cells were co-transfected with or without shGCH1 (pshGCH1) in the presence of pcDNA3-rGCH1 for two days. (b) Schematic illustration of rAAVs encoding GFP and shGCH1; GFP and shGCH1 expression cassettes were located in opposite directions to minimize interference with each other. (c) HeLa cells were co-treated with pcDNA3-rGCH1 and rAAV at the designated multiplicity of infection (MOI) for two days. Western-blotting analyses revealed a significant decrease in the rGCH1 levels in the presence of shGCH1. MT; mock-treated. ITR; internal terminal repeat of rAAV.

For knockdown of rGCH1 expression, nine siRNAs targeting various rGCH1 exon regions were designed (Fig. 1). The GCH1 silencing effect was examined using 293T cells exogenously expressing rGCH1, which were transfected with rGCH1-specific or control non-specific siRNA. Western-blotting analysis revealed that all specific siRNAs markedly inhibited rGCH1 expression, while control siRNAs did not. For further analysis, we selected a siRNA sequence targeting exon 4 of rGCH1 and generated a shGCH1 construct expressing shRNA under the control of a H1 promoter [15]. As expected, shGCH1 dramatically suppressed rGCH1 expression, similar to its corresponding siRNA (Fig. 2a). To achieve persistent shRNA expression, we constructed rAAV-shGCH1 by inserting this shGCH1 expression cassette into the rAAV2 backbone. rAAV-shGCH1 additionally encodes the GFP reporter gene driven by the CMV promoter to allow the simultaneous tracking of shGCH1 expression (Fig. 2b). rAAV-GFP lacking the shGCH1 expression cassette was employed as a control. The rGCH1 silencing effect was viral dose-dependent in HeLa cells (Fig. 2c). As expected, the rGCH1 level was not affected by the control virus. A similar degree of GFP expression was observed with equivalent amounts of either virus. Our data collectively suggest that rAAV-mediated shGCH1 synthesis effectively suppresses GCH1 expression.

### Delivery of rAAV-mediated shGCH1 into dorsal root ganglion (DRG)

**Figure 3:**
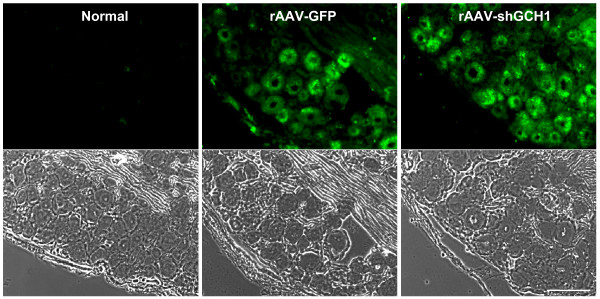
**Efficient gene delivery into DRG through sciatic nerve injection**. The left sciatic nerves of rats were injected with rAAV-GFP or rAAV-shGCH1. At 14 days after injection, rats were sacrificed and L5 DRGs removed following perfusion. DRG tissue sections were prepared at a thickness of 10 μm. Fluorescent green signals were identified as GFP expression in both virus-treated groups under a fluorescent microscope. Scale bar = 100 μm.

Recent studies propose that sciatic nerve injection of rAAVs is a promising strategy for delivery of therapeutic genes to DRG [21,22]. In view of these findings, we attempted to deliver transgenes into DRG by administering 6.0 × 10^7 ^of rAAV-shGCH1 or rAAV-GFP to the sciatic nerve 9 days after spared nerve injury (SNI). Gene transfer efficiency was confirmed by the presence of GFP-positive DRG neural cells under a fluorescent microscope (Fig. 3). Intense green signals were discerned in all infected mice from both experimental groups on day 14 post virus injection (n = 4 for each group), indicative of effective GFP transgene expression. In contrast, green fluorescence was absent in control groups (n = 5). In equivalent experiments involving treatment with viruses prior to pain surgery, we observed analogous patterns of notable GFP expression in experimental groups (data not shown). The data confirm that the sciatic nerve is a useful delivery route for transducing the DRG with the genes of interest, in both cases of pre- and post-injection.

### Attenuation of neuropathic pain behavior through downregulation of GCH1 levels via rAAV-shGCH1

**Figure 4:**
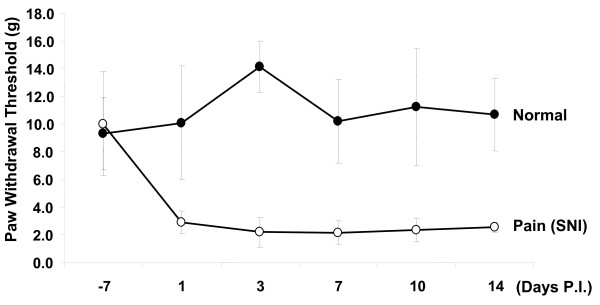
**Mechanical pain development following pain injury**. Spared nerve injury was operated by tightly ligating and transectioning the tibial and common peroneal nerves and leaving sural nerves intact. As shown here, the animals promptlly developed pain behavior that was determined by mechanical von Fray threshold.

**Figure 5:**
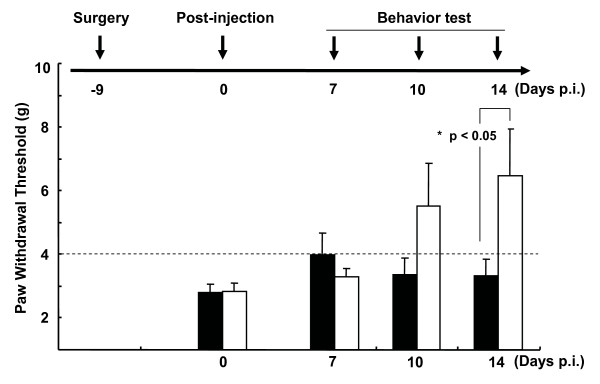
**Attenuation of neuropathic pain behavior upon post-injection with rAAV-shGCH1**. Pain hypersensitivity of the rats was measured using up-and-down methods with the von Frey filaments on the designated days. Values below the broken line (threshold = 4) represent pain-like behavior. Data are presented as means ± SEM (n = 10 for rAAV-shGCH1 and rAAV-GFP- injected pain groups; n = 6 for the normal control group). An asterisk (*) indicates that the rAAV-shGCH1-injected group significantly differs from the rAAV-GFP-injected group (*p < 0.05). black square, rAAV-GFP; open square, rAAV-shGCH1.

**Figure 6:**
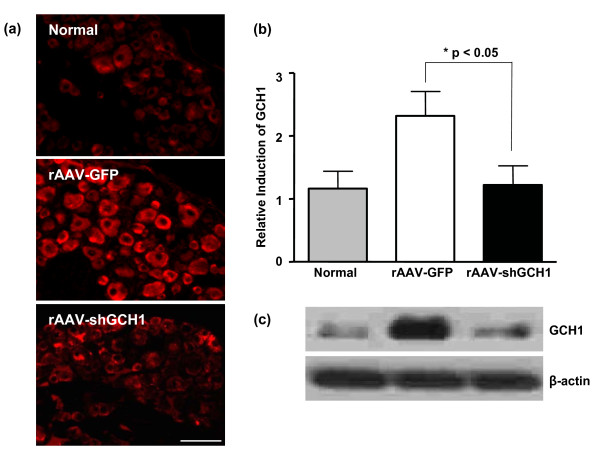
**Downregulation of the rGCH1 level by rAAV-shGCH1 in ipsilateral DRG**. Immunohistochemistry (a, b) and western-blotting analysis (c) using GCH1-specific primary antibody allowed the visualization of rGCH1 expression 14 days after virus administration. Secondary antibody conjugated with Alexa Fluor-555 was used. The relative intensity of GCH1 expression was quantified by counting immune-positive cells following immunohistochemistry (b). Data are presented as means ± SEM (n = 8 per each groups). *p < 0.05, Scale bar = 100 μm.

Spared nerve injury was generated by tight ligation and transaction of the tibial and common peroneal nerves while leaving sural nerves intact [23], and promptlly developed pain behavior which was determined by mechanical von Fray threshold. Following surgery, the mechanical threshold began to drop sharply from normal range to pain threshold levels (Fig. 4). All the animals showed pain symptoms within few days which were maintained thereafter (on day 7; 2.2 ± 0.9 at the threshold level). To explore the beneficial effects of GCH1 down-regulation on neuropathic pain, related behavior was initially examined under blinded conditions. After introducing rAAV-shGCH1 (n = 10) or control rAAV-GFP (n = 10) into rats (9 days post pain surgery), the mechanical von Fray test was performed at the designated time-points. As shown in Fig. 5, the average von Fray threshold was 2.8 ± 0.3 at the threshold level on day 9 after pain surgery. Following rAAV-shGCH1 treatment, the pain threshold gradually declined over time, except in the case of rAAV-GFP as expected. To ensure that GCH1 down-regulation was specifically promoted by rAAV-shGCH1, GCH1-specific immunohistochemistry and western-blotting assay was performed in ipsilateral L4/5 DRGs. In immunohistochemistry, shGCH1 introduction via rAAV2 significantly suppressed GCH1 expression to a similar level as the no-pain control group, while the up-regulation of GCH1 remained unchanged in the rAAV-GFP injected group (Fig. 6a), which supports previous report showing the positive association of pain symptoms with GCH1 activation [10]. The relative GCH1 levels were 2.3 ± 0.4 and 1.2 ± 0.3 in GFP- and shGHC1-treated pain groups, respectively (Fig. 6b). In addition, the immunohistochemical staining against GCH1 showed a prominent negative correlation with the mechanical threshold of respective animals (Spearman rho test, Correlation coefficient = -0.470), though there was no statistical significance mainly due to small sample numbers. Similar pattern of GCH1 expression was also observed in western-blotting analysis (Fib. 6c). These results collectively illustrate that rAAV-shGCH1 treatment of the sciatic nerve effectively suppresses GCH1 expression and leads to effective pain relief.

**Figure 7:**
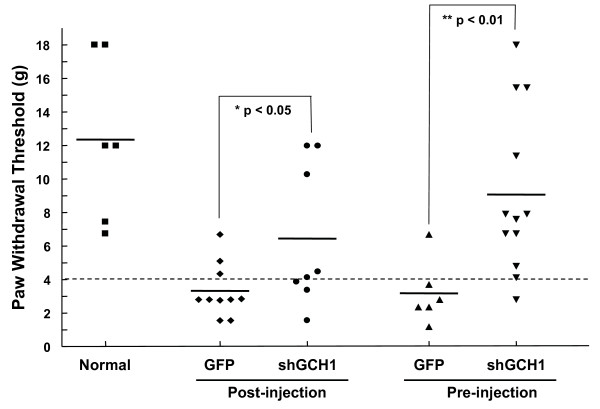
**Comparison of pain relief following pre- and post-injection of rAAV-shGCH1**. For pre-injection of rAAV, viruses were introduced into the sciatic nerve 7 days before surgery, and at 9 days after surgery. Pain behavior was estimated on day 14 after virus injection, as described in Fig. 3. Values below the broken line (threshold = 4) represent pain-like behavior. An asterisk indicates that the rAAV-shGCH1-injected group differs significantly from the rAAV-GFP-injected group (* p < 0.05; ** p < 0.001).

As predicted, injection of rAAV-shGCH1 prior to SNI prevented pain induction whereby several rats experienced no differences in the pain test compared to control animals (Fig. 7). Similar to post-injection, the anti-pain effect upon pre-treatment reached maximal levels on day 14 after virus injection. In conjunction with data obtained from Fig. 3, these results suggest that shGCH1 introduction into the sciatic nerve via rAAV2 delivery leads to marked attenuation of pain symptoms, regardless of treatment times.

### Suppression of inflammation activation by AAV-shGCH1

**Figure 8:**
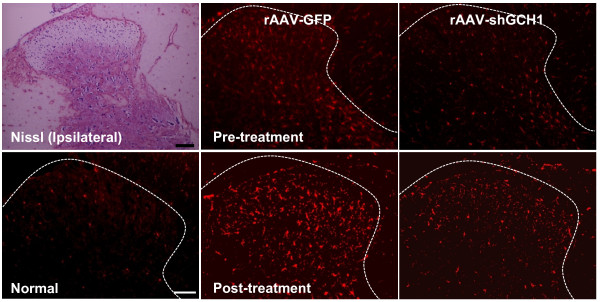
**Inhibition of microglial activation via rAAV-shGCH1 in the spinal cord dorsal horn**. Nissl-staining was carried out in the frozen sections of spinal cords. Microglia in the spinal cord dorsal horn were visualized with the Iba-1 primary antibody on day 14 after either pain surgery for pre-treatment and virus administration for post-treatment. Immunohistochemistry data reveal that the number of Iba-1 positive cells increased in the rAAV-GFP pain group only. Scale bar = 100 μm.

Previous studies have demonstrated that inflammatory activation of microglia in the ipsilateral spinal cord plays a crucial role in the development of neuropathic pain [24]. To determine whether GCH1 downregulation affects microglial activation, we performed immunohistochemistry with the Iba-1 antibody, a microglial marker (Fig. 8). Indeed, nerve injury activated microglia, as evident from both the increased number and the morphological changes of microglia in the ipsilateral dorsal horn (data not shown). The GFP pain group experienced similar microglial alterations. In contrast, microglial activation was significantly inhibited in the shGCH1 pain group, and a similar pattern of microglial inactivation was observed in case of virus pre-treatment. These results suggest that pain attenuation following shGCH1 introduction is associated with a decrease in inflammation.

## Discussion

The present study provides clear evidence of the therapeutic potency of GCH1 down-regulation in DRG via rAAV-shGCH1-mediated silencing for treating neuropathic pain. Initially, we confirmed that the sciatic nerve is an effective route for rAAV to transduce primary sensory neurons residing in ipsilateral DRG. shGCH1/siGCH1 was effectively synthesized in neural cell bodies of DRG and actively degraded GCH1 mRNA and disrupted protein synthesis. Decreased GCH1 expression was linked with the acquisition of pain resistance and decrease of microglial inflammation in the corresponding spinal cord.

GCH1 is the rate-limiting enzyme during the de novo synthesis of tetrahydrobiopterin (BH4), an essential cofactor for the formation of nitric oxide, biogenic amines and serotonin [8]. Hereditary diseases, such as DOPA-responsive dystonia and atypical phenylkenonuria, are attributed to GCH1 mutations [25]. However, recent evidence implies that genetic alterations in GCH1 have effects beyond 'loss-of function'-related symptoms. Among these, reduced pain hypersensitivity is evident [26,27]. GCH1 haplotypes comprising of 15 SNPs are associated with reduced pain symptoms [25], although conflicting reports are documented [28]. Our present data indicates that blockage of GCH1 over-expression effectively attenuates pain symptoms, strongly supporting previous findings on the close correlation between GCH1 down-regulation and pain relief.

Certain small molecules, such as the GCH1 inhibitor, 2-4-diamino-6-hydroxypyrimidien (DAHP), have been found to rapidly ameliorate pain symptoms in animal models [10]. However, DAHP usage in clinical situations is limited by its cytotoxicity and short-term effectiveness (lasting only a few hours). In this regard, rAAV-shGCH1 is superior as a novel therapeutic agent. shGCH1 can exclusively disrupt target GCH1 mRNA, facilitating a specific gene knockdown effect. Additionally, rAAV2 is an excellent gene delivery system for neural tissues [29]. Transduction of neural cells by rAAV is efficient and persistent with minimal toxicity. In our study, a single injection of the virus led to consistent down-regulation of GCH1 expression following pain surgery, and improved pain resistance over time. Thus, local synthesis of shGCH1 via rAAV in DRG may offer clinical benefits. The clinical potential of rAAV to treat several neurodegenerative diseases is currently under investigation in human trials [30,31].

One of the major advantages of gene-based therapy is its high efficacy and minimal side-effects, which is essentially achieved by the specific transduction of target cells/tissues in a regionally concentrated manner. Several studies have described the successful achievement of local gene expression in the DRG/dorsal horn through various routes [22]. The most well-established and direct technique is the administration of the vector into DRG itself. However, this method requires invasive surgery requiring the removal of a part of the spinal vertebra [19], which can result in unwanted nerve injury and side-effects. Intrathecal administration is an alternative method. However, its usefulness is limited by reduced effectiveness of gene transfer and poorer target specificity. In contrast, sciatic nerve injection is an attractive strategy [21]. Virus administration to the sciatic nerve is not as invasive as direct injection, and applicable in clinical situations. Consistent with other related studies, our data suggests that rAAV2-mediated gene delivery via the sciatic nerve effectively distributes the transgene to DRG, leading to successful acquisition of therapeutic activity with confirmed neuronal tropism. Hence, the sciatic nerve is an excellent route for local treatment of the DRG/spinal dorsal horn when rAAV is employed as a gene delivery vehicle.

As an additional advantage, we noted that shGCH1 treatment led to decreased activity of microglial cells which play a key role in inflammatory events associated with pain symptoms. Inflammatory events through the phosphorylation of signaling mediators in the p38 MAPK pathway are well documented during clinical pain development in various types of pain [24]. As key players in this event, microglial cells proliferate and undergo morphological alterations to their active forms in the ipsilateral dorsal horn of the spinal cord [32,33]. Microglial cells exhibit abnormally hyper activities--including phagocytic properties, secretion of neurotrophic factors, and cytokines--following the stimulation of the p38 phosphorylation signal within 3 days after nerve injury in an animal model [34,35]. Microglial activation, in turn, aggravates pain development and other related symptoms. Therefore, it is also vital to mitigate microglial inflammation for an effective pain cure. Interestingly, we observed that shGCH1 introduction significantly reduced microglial activation, regardless of the time-point after nerve injury. Finally, it is very interesting issue to verify long-term effects of GCH1 down-regulation especially focusing on modulation of microglial activation in spinal cord. In this regard, further studies are warranted to investigate the mechanism by which activated microglia in spinal cord might be associated with GCH1 expression and then participate in the initial development and/or persistence of pain. Efforts to exploring the inter-relationship between GCH1 expression and microglial activation may pave the way to develop new therapeutic modalities for neuropathic pain.

Based on these findings, we propose that: i) up-regulation of GCH1 following nerve injury is associated with inflammatory activation of microglia and development of pain; and ii) GCH1 down-regulation alters microglial activation patterns, and subsequently leads to relief of pain symptoms.

## Methods

### Cell culture

293T and HeLa cells were purchased from the American Type Culture Collection (Manassas, VA). Cells were maintained in Dulbecco's modified Eagle's medium (Gibco BRL, Carlsbad, CA) supplemented with 10% fetal bovine serum, L-glutamine (2 mM), penicillin (100 IU/ml) and streptomycin (50 μg/ml) at 37°C in a 5% CO_2 _incubator.

### siRNA preparation and treatment

All siRNAs against GCH1 were designed using software from MWG Biotech AG http://www.mwg-biotech.com. In total, nine duplex siRNAs (Supple. 1) with a dTdT 3' overhang and control scrambled siRNA were manufactured by DHARMACON (Lafayette, CL) with the "ready-to-use" option. Cells were transfected with 100 nM siRNA complexed with Oligofectamine reagent (Invitrogen, Carlsbad, CA) in OPTI-MEM medium (Invitrogen). After 3 h, media were replaced with growth medium containing 10% serum. The next day, cells were harvested and extracted for Western blotting.

### Construction of a bidirectional shRNA expression vector

The human H1 polymerase III promoter (pH1) for shRNA expression was amplified from HeLa cells using PCR-based methods, as described previously [36]. The shRNA sequences against rGCH1 (shGCH1) are 5'-GAT CCC GTG GAA ATC TAC AGT AGA A**TT CAA GAG A**TT CTA CTG TAG ATT TCC ACT TTT TTG GAA A-3' (sense) with a *Bam*HI linker and 5'-AGC TTT TCC AAA AAA GTG GAA ATC TAC AGT AGA **ATC TCT TGA A**TT CTA CTG TAG ATT TCC ACG-3' (anti-sense) with a *Hind*III linker. Nucleotides specific for rat GCH1 are underlined, and the loop structures presented in bold. The pH1-shGCH1 sequence was inserted adjacent to the CMV promoter of the vector expressing GFP, resulting in a bi-directional promoter (Figure 1a). All clones were verified by sequencing (data not shown).

### Preparation of rAAV containing shGCH1

To produce rAAV2, the viral backbone, pH1-shGCH1-pHpa-trs-SK, was co-transfected with pRepCaps and pXX6 (Stratagene, La Jolla, CA) encoding adenoviral helper genes [37,38]. For large-scale rAAV preparations, 293T cells were transfected in 20 × 10 cm dishes. rAAVs in cells were released and purified from cell lysates (including supernatant fractions) using two sequential CsCl gradient steps. After dialysis in 10 mM Tris buffer (pH 7.9) containing 2 mM MgCl_2 _and 2% sorbitol, rAAVs were aliquoted and stored at -80°C. The number of total virus particles was estimated with an ELISA kit (Progen Inc., Heidelberg, Germany) and real-time qPCR, as described in a previous report [38].

### Animal care and virus administration

Adult male Sprague Dawley rats weighing 180-200 g were housed with free access to food and water. With the approval of the University committee on the use and care of animals, rats were maintained in an optimal temperature and humidity-controlled room with a 12 hr light/dark cycle.

To inject rAAVs to the sciatic nerve, rats were anesthetized with Zoletil (50 mg/kg) and Rompun (10 mg/kg). Next, a segment of the left sciatic nerve was exposed at the mid-thigh level. Surrounding tissues were carefully removed under a surgical microscope, exposing sciatic nerve exposed (Olympus, Japan). A 3 μl viral solution containing rAAV-shGCH1 (6.0 × 10^6 ^viruses in 3 μl) or rAAV-GFP (4.0 × 10^6 ^viruses in 3 μl) was slowly injected into the nerves through a glass micropipette connected to a Hamilton syringe for 5 min. The pipette was pulled out after 5 min.

### SNI model and behavior test

To generate the spared nerve injury (SNI) model, the common peroneal and tibial nerves were tightly ligated and completely transected while the sural nerve was left intact [39]. Mechanical allodynia in rats was assessed by applying a series of Von Frey filaments to the plantar surface of the hind paw (range 0.01 - 1.66 g) in ascending and descending order (up-and-down method). Brisk withdrawal of the hind limb was considered a positive response. A withdrawal threshold of 4.0 g or less was classified as allodynia (pain-like behavior).

### Western- blotting

L4-5 DRG tissues were homogenized with lysis buffer containing 10 mM Tris-HCL (pH 8.0), 150 mM NaCl, 1% Triton-X100 and protease inhibitor cocktails (Sigma, St. Louis, MO), and centrifuged for 30 min at 4°C. Lysates (10-40 μg of proteins per lane) were separated by 10% SDS-PAGE and transferred onto PVDF membranes (Bio-Rad, Hercules, CA). Blots were incubated with the specified primary antibodies. The following antibodies were used: GCH1 (Abnova, Taiwan), β-actin (Sigma), and GFP (Santa Cruz Biotechnology, Santa Cruz, CA). Following incubation with peroxidase-conjugated anti-mouse (or anti-goat) IgG (Vector Laboratories, Burlingame, CA), proteins were detected using the chemiluminescence method (Pierce, Rockford, IL).

### Nissl staining & Immunohistochemistry

*Frozen tissue sections of 10 μm in thickness were prepared and Nissl-staining was carried out, as described previously *[40]. Immunostaining was also performed using frozen section. Briefly, after perfusion with 4% paraformaldehyde, DRG (L4, L5) and spinal cord (T13-L2) were removed and cryoprotected in 30% sucrose solution (dissolved in phosphate-buffered saline) for 24 h at 4°C before embedding in OCT compound (Sakura Finetek, Torrance, CA). Sections were incubated at 4°C with primary antibodies overnight. Antibodies used for immunohistochemistry were as follows: GCH1 (Abnova), NeuN (Chemicon, Temecula, CA), Iba-1 (Wako, Japan), phosphor-p38 (Cell signaling Technology, Beverly, MA), and OX-42 (Chemicon). Sections were subsequently incubated with Alexa 488-conjugated (or Alexa 555) secondary antibodies at room temperature for 60 min. After mounting using Vectashield with DAPI (Vector laboratories), images were observed by fluorescence microscopy (Leica, Germany).

### Statistics

All data were expressed as means ± SEM of three or more independent experiments. The statistical significance of differences between the values was determined by two-sample comparisons made using the 2-tailed *t *test or one-way ANOVA (analysis of variance) and post Dunnett's multiple comparison test. Statistical tests were performed using PRISM (GraphPad Software, San Diego, CA, USA). Differences were considered significant at P values less than 0.05.

## Conclusion

We have confirmed the therapeutic potential of shRNA-induced GCH1 down-regulation via rAAV in neuropathic pain treatment using the sciatic nerve as a delivery route. Consistent with previous reports showing beneficial effects on pain relief in patients with GCH1 genetic disorders, our findings strongly suggest that GCH1 modulation can be effectively developed as a clinically applicable gene therapy strategy to treat neuropathic pain.

## Competing interests

The authors declare that they have no competing interests.

## Authors' contributions

SJK, WIL and YSL carried out the studies described. HL and SWK supervised the experiments. DHK and JWC contribute to designing the present study and analyzing the results. SJK, HL, and SW Kim designed the experiments and wrote the manuscript. All authors read and approved the final manuscript.
